# Editorial: Diabetes in the Middle East

**DOI:** 10.3389/fendo.2021.638653

**Published:** 2021-02-19

**Authors:** Mohamed Abu-Farha, Jaakko Tuomilehto, Jehad Abubaker

**Affiliations:** ^1^ Biochemistry and Molecular Biology Department, Dasman Diabetes Institute, Dasman, Kuwait; ^2^ Department of Public Health, University of Helsinki, Helsinki, Finland; ^3^ Diabetes Research Group, King Abdulaziz University, Jeddah, Saudi Arabia

**Keywords:** Middle East, diabetes, obesity, metabolic syndrome, biomarker

## Introduction

Diabetes is a rapidly growing disease that is affecting people worldwide, particularly due to the rise in unhealthy lifestyles and increased risk factor levels. Although countries and regions are affected differently, the Middle East in general has witnessed a spike in the occurrence of diabetes. The Gulf region has experienced an especially marked and sudden increase in rates of diabetes where Kuwait, Saudi Arabia and Bahrain now rank among the top 10 countries with highest prevalence of type 2 diabetes worldwide (1). Additionally, children have also experienced a dramatic increase in rates of type 1 as well as type 2 diabetes in this region. Urgent action has been called for in order to tackle this problem in the Middle East, with the aim of reducing and preventing new cases of diabetes and complications due to diabetes.

This Research Topic has broadly focused on diabetes research in the Middle East, highlighting the increasing burden of the disease through recent epidemiological data. Weiderpass et al. conducted a cross-sectional study on Kuwaiti adults using the STEP-wise approach to surveillance of non-communicable disease risk factors. They updated the statistics for the prevalence of overweight and obesity in the population and found them to be 37% and 40.3%, respectively. Similarly, Djalalinia et al. conducted a national cross-sectional study of non-communicable disease risk factor surveillance in Iran. They found a significant difference between the prevalence of obesity in men vs. women (15.3 vs. 29.8%). They also report a considerable variation in the geographical pattern of the prevalence of obesity and overweight where BMI increased from the southeastern to the northwestern regions of the country. These findings show striking differences among the Middle East countries and the high burden of obesity affecting people in the Arabian Gulf region.

Our Research Topic also discussed childhood obesity where Goodson et al. conducted a longitudinal study on Kuwaiti children and performed salivary metabolomic analysis. They report that the level of salivary N1-Methyl-2-pyridone-5-carboxamide (2PY), a biomarker for uranium uptake, has the highest direct association with obesity. Elkum et al. performed a cross-sectional analysis on schoolchildren in Kuwait with Arab ethnicity, the prevalence of overweight and obesity was 17.7% and 33.7%, respectively. They also identified several predictors of childhood obesity including high birth weight, advanced maternal age at index pregnancy and small family size. Additionally, Saraswathi et al. presented a systematic literature review of childhood diabetes research in the Middle East Region. They reported that while many studies focused on the incidence/prevalence of different types of diabetes in childhood, there is a lack of consolidated studies focusing on national epidemiological data of childhood diabetes and also no studies reporting clinical trials in children with diabetes in the Middle East Region. Furthermore, Farran et al. performed a retrospective cohort study of health data from Kuwait to evaluate the use of non-invasive parameters and machine-learning algorithms for predicting future risk of type 2 diabetes. They found that machine-learning techniques such as k-nearest neighbors (k-NN) and support vector machines (SVM) outperformed the most used logistic regression methods.

This Research Topic also covered research conducted on diabetes-related pathways involving genomics, obesity, insulin resistance, inflammation dyslipidemia as well as diabetes complications. Qaddoumi et al. performed a retrospective study to evaluate metabolic control in patients with type 2 diabetes at Dasman Diabetes Institute, a specialist diabetes clinic and research center. They concluded that the therapeutic management of type 2 diabetes in Kuwait is suboptimal. They provide some recommendations such as better adherence to American Diabetes Association guidelines, evaluating the high obesity rates, as well as promoting diabetes education and self-empowerment. Azzam et al. investigated genetic variations associated with diabetic retinopathy and coronary artery disease in a case-control study conducted in UAE. They reported two SNPs to be associated with diabetic retinopathy (rs9362054 near *CEP162* and rs4462262 near *UBE2D1*) and rs12219125 near *PLXDC2* to be associated with coronary artery disease. They also reported rs9362054 near *CEP162* to be significantly associated with both diabetic retinopathy and coronary artery disease. Hebbar et al. present an extensive perspective on the possible causes for observed differences in the metabolic trait loci profiles between Europeans and Arabs. They also suggested analysis strategies and study designs that can be integrated for identifying genetic risk variants associated with diabetes and related traits in Arab populations. A cross-sectional study by Alghanim et al. investigated the expression levels of circulating ANGPTL5 protein in the circulation in people with obesity and type 2 diabetes and found that higher levels of ANGPTL5 in the circulation were associated with insulin resistance.

Al-Ozairi et al. investigated the feasibility of intermittent fasting (in the form of Ramadan fasting) in people with type 1 diabetes and concluded that intermittent fasting can be safe for patients with uncomplicated diabetes when combined with structured education and advanced glucose monitoring systems. Barda et al. investigated the impact of carbohydrate restriction and insulin treatment on placental maternal and fetal vascular circulation in obese and non-obese women with gestational diabetes mellitus. They reported that the combination of obesity and gestational diabetes increased the risk of fetal thrombo-occlusive disease and the occurrence of gestational hypertension. They also observed that carbohydrate restriction diet plus insulin treatment was associated with improved fetal placental vascular circulation. The study of Alajmani et al. assessing depression among people with type 2 diabetes in UAE found that the overall depression prevalence using a cutoff of 16 points in the Beck Depression Inventory was 17%. They concluded that the intensive service in a diabetes mini clinic compared to primary health care centers appears to benefit psychological aspects in diabetic patients.

In conclusion, our Research Topic covered multiple topics relating to obesity and diabetes in the Middle East. It highlighted the increased burden of these metabolic disorders especially in the Arabian Gulf region. It also showed the importance of enhancing diabetes research in the region, and the lack of structured early diabetes prevention and management programs that can aim at reducing the burden of diabetes and its associated complications needs to be developed urgently.

## Author Contributions

All authors have contributed equally to this work. All authors contributed to the article and approved the submitted version.

## Conflict of Interest

The authors declare that the research was conducted in the absence of any commercial or financial relationships that could be construed as a potential conflict of interest.
